# Income and Physical Activity among Adults: Evidence from Self-Reported and Pedometer-Based Physical Activity Measurements

**DOI:** 10.1371/journal.pone.0135651

**Published:** 2015-08-28

**Authors:** Jaana T. Kari, Jaakko Pehkonen, Mirja Hirvensalo, Xiaolin Yang, Nina Hutri-Kähönen, Olli T. Raitakari, Tuija H. Tammelin

**Affiliations:** 1 LIKES–Research Center for Sport and Health Sciences, Jyväskylä, Finland; 2 Jyvaskyla University School of Business and Economics, Finland; 3 Department of Sport Sciences, University of Jyvaskyla, Finland; 4 Department of Pediatrics, University of Tampere and Tampere University Hospital, Finland; 5 Research Centre of Applied and Preventive Cardiovascular Medicine, University of Turku and Department of Clinical Physiology and Nuclear Medicine, Turku University Hospital, Turku, Finland; Indiana University, UNITED STATES

## Abstract

This study examined the relationship between income and physical activity by using three measures to illustrate daily physical activity: the self-reported physical activity index for leisure-time physical activity, pedometer-based total steps for overall daily physical activity, and pedometer-based aerobic steps that reflect continuous steps for more than 10 min at a time. The study population consisted of 753 adults from Finland (mean age 41.7 years; 64% women) who participated in 2011 in the follow-up of the ongoing Young Finns study. Ordinary least squares models were used to evaluate the associations between income and physical activity. The consistency of the results was explored by using register-based income information from Statistics Finland, employing the instrumental variable approach, and dividing the pedometer-based physical activity according to weekdays and weekend days. The results indicated that higher income was associated with higher self-reported physical activity for both genders. The results were robust to the inclusion of the control variables and the use of register-based income information. However, the pedometer-based results were gender-specific and depended on the measurement day (weekday vs. weekend day). In more detail, the association was positive for women and negative or non-existing for men. According to the measurement day, among women, income was positively associated with aerobic steps despite the measurement day and with totals steps measured on the weekend. Among men, income was negatively associated with aerobic steps measured on weekdays. The results indicate that there is an association between income and physical activity, but the association is gender-specific and depends on the measurement type of physical activity.

## Introduction

The detrimental health consequences of physical inactivity are well-established [1–6]. Physical inactivity has been identified as the fourth-leading risk factor for global mortality [7], and several studies have reported increasing healthcare costs due to physical inactivity [6, 8–11]. Nevertheless, a substantial part of the population is not physically active [11–12].

The empirical literature that has examined the relationship between economic determinants and physical activity is large and yet increasing [13–22]. In general, the evidence suggests a positive association between an individual’s economic resources and physical activity. Meltzer and Jena [22], for example, found a positive association between income and self-reported participation in physical activity. People in the highest income group tended to have a 26% higher exercise energy expenditure and a 3% higher exercise intensity than those in the lowest income group. Similarly, Humphreys and Ruseski [18–19] found a positive association between income levels and participation in physical activity. Individuals with higher incomes were more likely to participate in any type of physical activity than those with lower incomes. Humphreys and Ruseski [20] also implied that income is an important determinant of physical activity: Individuals with higher incomes were more likely to participate in physical activities, but depending on participation, individuals spend less time on physical activities. Brown and Roberts [13], in turn, contended that the marginal effects of non-labor income and hourly wage on participation in physical activity may be relatively small. Moreover, the results suggested that monetary subsidies to promote participation in physical activity by working individuals may lead to a less than 1% increase in the frequency of participation in physical activity compared to the base category with no physical activity [13].

However, the relationship between income and participation in physical activities can be more complex than expected. This view is based on the simple assumption of utility maximization. In short, given time and budget constraints, individuals allocate their time in order to maximize a given utility function comprising the consumption of commodities and leisure [23]. This behavior suggests that although higher incomes provide more opportunities for physical activity (the income effect), higher incomes may also increase the opportunity cost of leisure time and, as a result, decrease the amount of time spent on such activities (the substitution effect). According to Special Eurobarometer on Sport and Physical Activity [24], the main barrier to physical activity was lack of time (42%). Additionally, McConnell’s [25] and Humphreys and Ruseski’s [18, 20] models are examples in which the effect of changes in income and the opportunity cost of time have opposite effects on the time spent on physical activity and overall participation in physical activity. Melzer and Jena [22] and Brown and Roberts [13] provided two recent empirical contributions that support this view. These studies indicated that higher incomes may lead to more intense physical exercise [22] as well as decrease the overall intention to exercise [13]. Economy-level income measures provided similar findings. Ruhm [14] indicated that physical activity decreased when the economy strengthened, and higher joblessness was related to higher levels of physical activity. In general, Ruhm [14] illustrated that a temporary deterioration in the economy was associated with health improvements.

In recent years, a growing number of population-based studies have used objectively measured physical activity (e.g., pedometers and/or accelerometers) [12, 26–29], apart from self-reported questionnaires. However, to our knowledge, studies that investigate the relationship between income and physical activity by using self-reported as well as objective measures of physical activity have not been conducted. In a broader context, a better understanding of how income is associated with different dimensions of physical activity can aid health promoters in implementing efficient tools for increasing participation in physical activity for individuals with different socioeconomic backgrounds. The purpose of the present study was to examine the associations between an individual’s income and physical activity in adulthood by using data from the Young Finns Study (YFS). In particular, the study extended the previous literature by using three measures of physical activity: the self-reported leisure-time physical activity index, pedometer-based daily total steps, and pedometer-based aerobic steps reflecting continuous activity for more than 10 min at a time. We hypothesized that income is positively associated with physical activity.

## Materials and Methods

### Ethics Statement

The study was approved by the local ethics committees (The Ethics Committee of the Hospital District of Southwest Finland), and each participant gave written informed consent before participating in the study.

### Study Population

The data were drawn from the ongoing longitudinal YFS, which was launched in 1980 [30]. The population of the YFS consisted of a random sample of boys and girls from six age cohorts (3, 6, 9, 12, 15, and 18 years at the baseline year in 1980) from five university towns in Finland with medical schools (Helsinki, Turku, Tampere, Oulu, and Kuopio) and the surrounding rural areas. In 1980, of the 4326 invited individuals, 3596 participated in the baseline study. The study has been conducted in seven follow-up phases (1983, 1986, 1989, 1992, 2001, 2007, and 2011). The examinations have included comprehensive data collection using questionnaires, physical measurements, and blood tests. The most recent follow-up was performed in 2011, when 2060 of the original participants, aged 34 to 49 years, participated in the examinations. Valid self-reported and pedometer-based physical activity details were obtained from 1155 (715 women and 440 men) individuals. Of those, 753 individuals (mean age 41.7 years; 64% women) were included in the present study sample. In 2008, a detailed description of the cohort profile was formed [30]. Overall, participation has been evolved over time. Some of those who were lost to follow-up early in the study returned later. Those who dropped out were more often men and younger than those who remained in the study. A comparison of the physical activity levels showed no difference between the participants and the dropouts [31].

### Physical Activity

Three variables illustrated daily physical activity in 2011: the self-reported leisure-time physical activity index (PAI), total steps per day, and aerobic steps per day. Self-reported leisure-time physical activity, expressed as the PAI, ranging from 5 to 15, was collected with a questionnaire. The PAI was formed as the sum of five variables that describe the frequency and intensity of physical activity, the frequency and average duration of moderate to vigorous physical activity sessions, and participation in organized physical activity during leisure time [32]. Physical activity was also measured with a pedometer (Omron Walking Style One HJ-152R-E) for 7 consecutive days [31], and the results were expressed as total steps per day and aerobic steps per day. Total steps per day contained every step that was taken during the day including leisure time and working time. Since physical activity recommendations suggest that for optimal health benefits a minimum of moderate intensity aerobic physical activity should be performed in periods of at least 10 min [7, 33], the aerobic steps were calculated automatically for continuous walking ≥10 min without interruption at a pace of >60 steps/min. The steps measured with the Omron Walking Style pedometer were comparable to the steps measured with the ActiGraph accelerometer (GT1M) with a correlation coefficient of 0.942 (P < 0.001) [31].

### Income

The information on an individual’s annual income was collected with a self-reported questionnaire in 2007 and 2011 and information on household income in 1980, 1983, and 1986. From those years, three years (1980, 2007, and 2011) were chosen in the present study to illustrate an individual’s income. In 2011, the variable contained 13 response categories, and in 2007 eight categories. In the baseline year 1980, household income contained eight categories, and the values were converted into euros by using monetary value coefficients from Statistics Finland.

### Statistical Analysis

STATA SE/13.1 for Windows was used for the statistical analyses. Correlation coefficients between the measures of physical activity were calculated. In addition, the following ordinary least squares (OLS) regression model was formed to explore the association between income and physical activity:

$$PHYSICAL\;ACTIVITY_{ijt}=\alpha_{i}+\beta \times INCOME_{ijt}+\mu \times X_{ijt}+\varepsilon_{ijt},$$ (1)

where the subscript *i* describes the individual, *t* describes the study year, and *j* captures the alternative physical activity dimensions: the physical activity index [PAI_*it*_], total steps per day [STEPS_*it*_], and aerobic steps per day [ASTEPS_*it*_]. α_i_ indicates the unobserved but time-invariant differences in physical activity between individuals, and ε_*ij*_ is the stochastic error term with constant variance. β is the main parameter of interest, measuring the association between physical activity and income at the given vector of the control variables (X_*it*_). The data provided several possible controls (i.e., potential confounding factors), consisting of observed socioeconomic characteristics (age, neighborhood, marital status, number of children, years of education, work status [working/not working], and workload), health status (summary of the self-reported number of diseases and body mass index [BMI]), and family background factors (parental education and parental physical activity measured in 1980). Information on family background factors from the baseline year 1980 provided controls for unobserved heterogeneity, such as innate ability and preferences, thus alleviating possible biases in the estimated correlation with physical activity and income.

The consistency and robustness of the OLS estimator of our model (Eq 1) require that the income variable and the observed control variables are uncorrelated with the error term (ε_*ijt*_) and that unobserved individual heterogeneity is uncorrelated with the income variable. We supported as well as to scrutinized the robustness of these assumptions with the following ways. First, following Angrist and Picshke [34], we used predetermined values for all observable controls (X_*it*_). Assuming that individuals do not make forward-looking plans for physical activity, future physical activity cannot have an effect on the control variables measured before the level of physical activity is chosen. Therefore, future physical activity cannot have an effect on the control variables measured before physical activity. Second, we controlled unobserved individual heterogeneity by using data on individuals’ family background; that is, we controlled parental education and parental physical activity observed in the baseline year 1980.

## Results and Discussion

### Sample Attrition

Table 1 presents the mean and standard deviations of the study sample, N = 753 according to gender (see the comparisons of the full sample and the study sample from S1 Table). The size of the full sample varied depending on the number of missing values (S1 Table). For example, valid self-reported and pedometer-based physical activity details were observed from 1155 (N = 715 women and N = 440 men) individuals. The study sample locked up the number of individuals (N = 753), where all subjects with missing observations were excluded from the analysis. This enables the comparison between the models. Although the differences between the samples were modest, some features required attention. First, the means of physical activity measurements were higher in the study sample than in the full sample (P ≤ 0.001–0.025), except the self-reported physical activity among men. Second, there were no differences in the income means in the study sample compared to the full sample.

**Table 1:** Descriptive statistics of the study sample N = 753 (N = 479 women and N = 274 men).

Variable	Women		Men		P-value^a^
	Mean	SD	Mean	SD	
**Physical activity**					
Physical Activity Index (PAI)^b^	9.36	1.77	8.98	1.83	0.005
Frequency of PA	1.92	0.01	1.86	0.02	0.018
Intensity of PA	2.12	0.02	2.20	0.03	0.012
Duration of PA	2.09	0.02	2.16	0.03	0.041
Frequency of MVPA	1.66	0.02	1.63	0.03	0.401
Organized sports	1.51	0.03	1.16	0.02	<0.001
Total Steps / Day	8865	2811	8101	2874	<0.001
Aerobic Steps / Day	2789	2174	2005	2004	<0.001
**Socioeconomic characteristics**					
Age (years)	41.75	4.98	41.68	5.12	0.837
Income^c^	6.72	2.69	8.63	3.10	<0.001
Education (years)	16.27	3.31	15.50	3.33	0.002
Work status^d^	0.92	0.28	0.98	0.16	<0.001
Light sedentary work	0.34	0.47	0.33	0.47	0.911
Heavy physical work	0.01	0.07	0.02	0.12	0.184
Number of children	1.75	0.44	1.69	0.46	0.101
Married	0.79	0.41	0.83	0.38	0.153
Suburb	0.49	0.50	0.49	0.50	0.956
**Health status**					
Number of diseases^e^	1.13	1.23	0.85	1.02	0.001
Body Mass Index	24.76	4.42	26.17	3.96	<0.001
**Family background factors**					
Education (years) Mother	10.06	3.05	10.27	3.31	0.381
Education (years) Father	9.90	3.81	9.88	3.79	0.944
Physical activity Mother^f^	1.67	1.51	1.62	1.48	0.698
Physical activity Father^f^	1.88	1.70	1.81	1.61	0.565

PA, physical activity; MVPA, moderate to vigorous physical activity.

^a^ P-values for gender differences (T-test).

^b^ Physical Activity Index (PAI) is a summary of five variables that illustrate the frequency and the intensity of physical activity, the average duration of physical activity sessions, the frequency of moderate to vigorous physical activity sessions, and participation in organized sports during leisure time. Each response alternatives were coded from 1 to 3, and thereafter added up to form a PAI with a scores ranging from 5 to 15.

^c^ Income categories: 1 = < €5000, 2 = €5000–10000, 3 = €10001–15000, 4 = €15001–20000, 5 = €20001–25000, 6 = €25001–30000, 7 = €30001–35000, 8 = €35001–40000 9 = €40001–45000, 10 = €45001–50000, 11 = €50001–55000, 12 = €55001–60000, 1 3 = > €60000.

^d^ Dummy-variable, which gets value 1 if working, and value 0 if not working.

^e^ Self-reported number of diseases.

^f^ Self-reported parental physical activity obtained in 1980. The question contained the frequency of physical activity (1 = Never, 2 = Once a month, 3 = 2–3 times/month, 4 = Once a week, 5 = 2–6 times/week 6 = Daily).

One general feature of the data was that women tended to be more physically active compared to men based on all three physical activity measures (Table 1). However, this is in line with national studies that have examined physical activity levels among Finnish adults [35]. In addition, interesting details were found when the focus was on the type of the self-reported physical activity (Table 1). First, the average duration (P = 0.041) as well as the intensity (P = 0.012) of physical activity sessions were higher among men, whereas women participated more often in organized sports (P < 0.001). Second, the frequency of physical activity was higher among women compared to men (P = 0.018). Finally, no differences were found in the frequency of vigorous physical activity. Based on the previous studies, in turn, men were typically more active compared to women [13, 18–20, 21–22]. For example, men tended to exercise longer, more intensively [22], and more frequently [13] and had higher tendency to participate in physical activity [21] compared to women.

### Preliminary Results

The variation in three physical activity measures with income tertiles (Low, Middle, and High) is described in Table 2. The self-reported physical activity varied significantly with income for both sexes (P = 0.001 and 0.027); individuals with higher income had a higher PAI. However, total steps per day varied by income only in men (P = 0.004). Men with higher income had fewer mean total steps per day; that is, men in the high-income group had on average 1400 fewer steps than those in the low-income group. Aerobic steps, in turn, varied significantly with income only in women (P = 0.008). Women in the high-income group had on average 460 more aerobic steps per day than those in the low-income group.

**Table 2:** Summary statistics: Physical activity level with income tertiles: Low, Middle, and High^a^ among women (N = 479) and among men (N = 274).

Income Group	Self-reported Physical Activity		Pedometer-based Physical Activity			
	Physical Activity Index (PAI)		Total Steps		Aerobic Steps	
	Women	Men	Women	Men	Women	Men
Low	9.02 (1.71)	8.61 (1.78)	8566 (2988)	8707 (3124)	2328 (1879)	2159 (2024)
Middle	9.30 (1.75)	9.05 (1.67)	9038 (2696)	8193 (2574)	2948 (2370)	1998 (1948)
High	9.77 (1.78)	9.32 (1.99)	8947 (2764)	7311 (2701)	3049 (2133)	1835 (2044)
F-test	6.92	3.68	1.24	5.75	4.93	0.60
P-value^b^	0.001***	0.027**	0.289	0.004***	0.008***	0.547

Robust standard errors are in the parentheses.

^a^ Income divided into tertiles: Low, Middle, and High by gender. Each group contains a third of the study sample.

^b^ Significant at **5%, and ***1% level.

The correlation analysis (Table 3) illustrates the associations between the measures of physical activity. In general, the correlation coefficients were higher in women than in men. Moreover, as in the study by Tudor-Locke et al. [36], the correlation coefficient between the self-reported PAI and pedometer-based measures depended on how the pedometer outputs were expressed (e.g., total or aerobic steps), that is, the correlations were stronger between the PAI and aerobic steps compared to those of the PAI and total steps. Since the correlations between the self-reported and pedometer-base measures were modest, the results suggest that each physical activity variable may illustrate a different dimension of physical activity: The PAI reflects leisure-time physical activity, total steps the overall daily physical activity, and aerobic steps continued and intensive activity that lasts more than 10 min.

**Table 3:** Correlation between different physical activity measures among women (N = 479) and among men (N = 274).

	Physical Activity Index	Total Steps	Aerobic Steps
**Women**			
**Physical Activity Index**	1.000		
**Total Steps**	0.213***	1.000	
**Aerobic Steps**	0.303***	0.712***	1.000
**Men**			
**Physical Activity Index**	1.000		
**Total Steps**	0.138***	1.000	
**Aerobic Steps**	0.286***	0.575***	1.000

*** Significance at the 1% level.

### OLS Results

Table 4 reports the OLS estimates for alternative physical activity measures and two alternative model specifications. For the PAI, the income coefficient measured the impact of a unit change in income level on the index of self-reported physical activity. Total steps and aerobic steps were expressed in natural logs, so the income estimate depicted a percent change in steps. To ensure comparisons between the specifications, all individuals with missing values were excluded from the analysis, and the total number of observations used in the analysis was 753.

**Table 4:** Regression of the self-reported physical activity index and the pedometer-based physical activity (total steps and aerobic steps), study sample (N = 753), women (N = 479), men (N = 274).

	Self-reported physical activity		Pedometer-based physical activity			
	Physical activity index		Total steps		Aerobic steps	
	Model 1	Model 2	Model 1	Model 2	Model 1	Model 2
**Women**						
Income	0.11*** (0.029)	0.11*** (0.033)	0.01* (0.006)	0.01** (0.006)	0.05*** (0.016)	0.04** (0.017)
R^2^	0.03	0.08	0.01	0.07	0.03	0.07
**Men**						
Income	0.11*** (0.034)	0.09* (0.047)	–0.02*** (0.007)	–0.01 (0.008)	–0.02 (0.020)	–0.04* (0.025)
R^2^	0.04	0.12	0.04	0.16	0.01	0.11
**Control variables**	No	Yes	No	Yes	No	Yes
Socioeconomic Characteristic	-	x	-	x	-	x
Health Status	-	x	-	x	-	x
Family Background factors	-	x	-	x	-	x

Significant at *10%

**5%, and

***1% level.

Robust standard errors are in the parentheses.

Total steps and aerobic steps are transformed with natural logs.

Added control variables: the vector of socioeconomic characteristics (neighborhood, marital status, number of children, years of education, work status, and physical workload), health status (self-reported number of diseases and BMI) observed in 2007, and family background factors (parental education and parental physical activity) observed in 1980.

According to the results (Table 4), higher income was associated with higher physical activity in women. The relationship was statistically significant and robust across alternative physical activity measures as well as the inclusion of control variables. Having a one-unit higher income level was associated with a 0.11-unit higher PAI, approximately 1% more total steps, and approximately 4% more aerobic steps. Similarly, in men, the results indicated that higher income was related to higher self-reported physical activity. The income estimate was robust regarding the inclusion of control variables. However, unlike as hypothesized, for the pedometer-based measures (total steps or aerobic steps), the estimates were negative, and their statistical significance depended on the model specifications. In the case of total steps, the inclusion of observable controls made the income estimate statistically insignificant. For aerobic steps, the inclusion of controls, in turn, provided a negative and statistically significant estimate. Therefore, in men, having one unit higher income was associated with higher self-reported physical activity but a lower number of total and aerobic steps.

The results in case of the self-reported physical activity were consistent with the previous literature, which has suggested income as a determinant of physical activity, and that the association between the variables is positive [13, 15, 19, 21, 22, 37]. In agreement with our results, Brown and Roberts [13] showed that the magnitude of income was similar among women and men. Eberth and Smith [21], in turn, showed that the magnitude of household income was slightly higher among men, whereas in studies conducted by Farrell and Shields [15], Humphreys and Ruseski [19], and Meltzer and Jena [22], gender differences were not analyzed. In addition, the OLS results were in line with the study by Brown and Roberts [13], which reported a relatively small association between income and participation in physical activity. According to the present study, one-unit higher income was associated with a 0.09–0.11 higher mean PAI value. The estimates implied that the difference between the mean PAI in the lowest (< €5000) and in the highest (> €60000) income categories was 1.08 to 1.32 units, depending on the model specifications. The explanatory power (R^2^) of the models varied between 0.01 and 0.16. This implies that although the results demonstrated an association between income and physical activity, in overall, the magnitude of income in predicting physical activity is relatively modest. Nevertheless, the results were in line with the previous literature; see, for example, studies by Eberth and Smith [21], R^2^ = 0.07 and Humphreys and Ruseski [19], where the R^2^ varied between 0.04 and 0.09.

The pedometer-based results were less clear-cut in men. Limitations related to pedometer-based measurements are known, and part of the inconsistency between the self-reported and objectively measured results may be due to the pedometer method itself. Although pedometers are a relatively simple and affordable method for measuring physical activity [36], they are not designed to distinguish the intensity of physical activity. However, because steps were divided into total steps per day and aerobic steps per day from which aerobic steps reflected continuous walking that lasted for more than 10 min at a pace of 60 steps/min, we may assume that aerobic steps represents a more intense exercise type of physical activity than total steps. In addition, pedometers are not sensitive to non-ambulatory activities such as swimming, gym workouts, cycling, or similar activities. As shown in Table 1, the type of physical activity may also be gender-specific; that is, women and men may prefer different types of physical activity. Therefore, one possible explanation for the inconsistent results may be the type of physical activity itself. For example, Finnish women typically participate more often in walking, cycling, and aerobics/gymnastics, whereas men participate more often in running, ball games, and gym training [38]. Finally, men and women may also under- or overestimate their self-reported levels of physical activity [39], which, may be related to health-enhancing awareness [40]. Therefore, the mismatch between the self-reported and the pedometer-based results may reflect a possible exaggeration of the self-reported measurements.

### Sensitivity Analysis

The OLS results suggested that the estimate of the association between physical activity and income did not suffer from omitted variable bias: The inclusion of a comprehensive set of control variables kept the income estimate by and large intact. However, there are other issues that may bias the results and tamper our conclusions. First, the association between income and physical activity might be spurious, and stemmed from unobserved factors that correlate with income and physical activity. An individual’s personality and ability are such factors. However, these variables are hard to come by, and typically, unobserved heterogeneity is accounted for by using panel data and fixed-effects models. In our case, the individual income measures obtained in 2007 and 2011 were based on different categorization, and therefore ruined the possibility of using fixed effects-models. Second, the findings may be biased by reverse causality. As several earlier studies have shown, physical activity has a positive impact on labor market returns. According to Long and Caudill [41], Ewing [42], and Stevenson [43], the labor market returns of former high school athletes were higher than those of their non-athlete counterparts. Similarly, Kosteas [44] showed that frequent exercise was associated with a 6% to 10% increase in wages, and Lechner [45] and Hyytinen and Lahtonen [46] reported positive long-term labor market effects in terms of monthly earnings and hourly wages. Therefore, if physical activity is a determinant of income, then estimates might reflect two-way causality, the impact running from physical activity to income. Third, the variables obtained with a self-reported questionnaire may contain errors that bias the coefficients [39].

The consistency of the OLS results was tested several ways. First, we used register-based earnings from Statistics Finland, to scrutinize the potential misclassification in self-reported income. Second, we used the instrumental variable (IV) approach to alleviate possible omitted variable bias and measurement error. Finally, the pedometer-based physical activity was divided according to the measurement day. This enabled us to test the possible role of time constraint; that is, we examined whether the association varied according to the day physical activity was measured.

To alleviate possible misclassification in the self-reported income measures, the physical activity details from the YFS were combined with the register-based Finnish Longitudinal Employer-Employee Data (FLEED) from Statistics Finland. The FLEED records detailed information on labor market variables over the period from 1990 to 2010. The link was based on unique personal identifiers and therefore avoided problems created by errors in record linkages [47]. In the combined data, YFS + FLEED, income referred to the average of the annual wages and salaries in 2010, where the mean income was €22504 among women, and €27617 among men (P < 0.001). In order to ensure the comparison between the register-based and self-reported income measures, the register-based income details were divided into 13 categories (see footnote c in Table 1). Moreover, only the baseline models (in correspondence to Table 4, Model 1) were formulated. One general advantage in the combined data was that income details were obtained for each participant with physical activity details (N = 1155) and therefore did not contain missing information.

As a result (Table 5), the estimates were consistent with the corresponding baseline results (see Table 4, Model 1). Among women, the association between income and physical activity was positive despite of the measurement type of physical activity. Amon men, as before, the sign and the significance of the association varied according to the measurement type of physical activity. In overall, the estimates were smaller than the corresponding baseline results obtained with the self-reported income measures.

**Table 5:** Regression of the register-based income^a^ and physical activity measures in women (N = 715) and men (N = 440).

	Self-reported Physical Activity	Pedometer-based Physical Activity	
	Physical Activity Index (PAI)	Total Steps	Aerobic Steps
**Women**			
Income	0.08*** (0.024)	0.01* (0.005)	0.04*** (0.013)
R^2^	0.01	0.01	0.02
**Men**			
Income	0.06** (0.026)	-0.01** (0.006)	-0.002 (0.015)
R^2^	0.02	0.02	0.01

Significant at *10%

**5%, and

***1% level.

Robust standard errors are in the parentheses.

Total steps and aerobic steps are transformed with natural logs.

^a^ Income information based on FLEED from Statistics Finland. The income referred to the average of the annual wages and salaries in 2010 and was divided into 13 categories (see footnote c in Table 1).

The results from the combined data provided evidence that the self-reported income measure did not suffer errors created by misclassification. However, to alleviate the possible measurement error in other variables, omitted variable bias, and test the robustness of the OLS results, we employed the IV estimation method [48]. The IV approach exploits the variation in income generated by a factor that, holding other things constant, affects only physical activity through income. The model requires two conditions to hold. First, the instrument needs to correlate with the income, and second, the instruments should be uncorrelated with the error term of the Eq (1). Our data included two possible instruments: income measured in 2007 and household income measured in the baseline year 1980.

In general, the IV results (Table 6) were consistent with the baseline OLS results suggesting a positive and statistically significant association between income and self-reported physical activity for both genders. However, the IV estimates for the self-reported physical activity were slightly larger than the corresponding OLS estimates. This suggests that the observed association between income and physical activity is not driven by omitted variables such as personality, ability, and unobserved family background factors [34]. In the case of pedometer-based measurements, only the unconditioned baseline specification (Model 1) suggested a statistically significant, and negative, association between income and total steps in men. Among women, in line with the OLS results, higher income was associated with an increasing number of aerobic steps.

**Table 6:** IV approach, study sample (N = 753), women (N = 479), men (N = 274).

	Self-reported physical activity		Pedometer-based physical activity			
	Physical activity index		Total steps		Aerobic steps	
	Model 1	Model 2	Model 1	Model 2	Model 1	Model 2
**Women**						
Income	0.15*** (0.041)	0.14*** (0.050)	0.01 (0.008)	0.01 (0.010)	0.07*** (0.021)	0.06** (0.025)
**Summary of the first-stage statistics**						
Sargan statistic	0.12	0.05	1.61	0.30	1.21	0.03
*p*-value	0.725	0.826	0.205	0.583	0.271	0.867
First-stage F	166.07	102.45	166.07	102.45	166.07	102.45
**Men**						
Income	0.12*** (0.042)	0.12** (0.062)	–0.03*** (0.008)	–0.01 (0.011)	–0.01 (0.024)	–0.02 (0.033)
**Summary of the first-stage statistics**						
Sargan statistic	4.85**	0.66	2.04	1.79	4.67**	1.39
*p*-value	0.028	0.418	0.153	0.181	0.031	0.238
First-stage F	287.28	170.18	287.28	170.18	287.28	170.18

Significant at **5%, and

***1% level.

Heteroskedasticity-robust standard errors are shown in the parentheses.

Total steps and aerobic steps are transformed with natural logs.

Two model specifications, one without control variables, excluding age (Model 1), and one with full set of control variables (Model 2). Added control variables: the vector of socioeconomic characteristics and health status observed in 2007, and family background factors observed in 1980.

Instruments used: Income, obtained in 2007 and household income obtained in 1980.

Because the Sargan test is not available with cluster robust standard errors, the test was executed with non-robust errors. Thus, the results should be treated with a care.

The pedometer-based results indicated that the relationship between income and physical activity may be more complex than previous studies, which have used only self-reported measures of physical activity, have suggested [49]. According to the Special Eurobarometer on Sport and Physical Activity [24], lack of time has been reported as the main barrier to physical activity among Finnish adults (32%). In addition, Meltzer and Jena [22] speculated the role of time as an important determinant of exercise patterns. Similarly, in the present study, the negative sign of income in men suggests that there may be a trade-off between work and physical activity. The time constraint may be more important than the budget constraint. Men with higher income had more self-reported physical activity during leisure time, but fewer daily total steps and continuous (>10 min) physical activity (aerobic steps) compared to those with lower income.

To test the role of time constraint, the baseline models were extended by dividing the pedometer-based physical activity on weekdays and weekends, assuming that on weekends working individuals may have more time to spend on physical activities. The results are presented in Table 7. The findings confirmed our previous results with some interesting details. Among women, income was positively associated only with the total steps measured during the weekend. Having a one-unit higher income level was associated with approximately 2% more total steps during the weekend. Income, in turn, was positively associated with aerobic steps, despite the measurement day, such that having a one-unit higher income level was associated with approximately 4–8% more aerobic steps. Among men, income was negatively associated only with aerobic steps measured on weekdays. In more detail, having a one-unit higher income level was associated with approximately 5% fewer aerobic steps on weekday.

**Table 7:** Regression of the pedometer-based physical divided into weekdays and weekend days, study sample (N = 753), women (N = 479), men (N = 274).

	Total Steps		Aerobic Steps	
	Weekday	Weekend day	Weekday	Weekend day
**Women**				
Income	0.01 (0.007	0.02** (0.010)	0.04** (0.018)	0.05*** (0.017)
R^2^	0.01	0.01	0.01	0.03
**Men**				
Income	–0.02 (0.010)	-0.01 (0.011)	–0.05** (0.024)	–0.01 (0.024)
R^2^	0.04	0.01	0.03	0.01

Significant at **5%, and

***1% level.

Robust standard errors are in the parentheses.

Total steps and Aerobic steps are transformed with natural logs.

Added control variables: the vector of socioeconomic characteristics and health status observed in 2007, and family background factors observed in 1980.

### Summary of the Results

The results provided evidence about the relationship between income and physical activity. In the case of self-reported measures, the association was positive despite the model specifications and the inclusion of control variables. The pedometer-based results, in turn, were gender-specific and depended on the measurement type of physical activity and the day physical activity was measured.

The results suggest a possible role for time constraint. This was particularly marked in case of pedometer-based results when the steps were divided into weekdays and weekends. Among women, income was positively associated with the total steps measured during the weekend, and among men income was negatively associated with the aerobic steps measured on weekdays. Therefore, among men, higher incomes may increase the opportunity costs of leisure time and, as a result, reduce the time spent on such activities, that is, the time constraint becomes more important than the budget constraint. However, to confirm this, more research is needed. Therefore, for future research, it would be valuable to divide daily physical activity into leisure time, occupational, and commuting physical activity. Moreover, analysis consisting of the types of physical activity, measured with self-reported and objective measures, would extend the current knowledge about the association between income and physical activity. Are there types of physical activity preferred by individuals with lower/higher income? Is this association gender-specific? Then the interplay between the opportunity costs and income effects could be explored in more detail. This information would also be useful to employers when they target workplace physical activity programs for workers with limited leisure-time resources. Furthermore, this information would benefit policy makers in order to implement effective methods for increasing overall daily physical activity for individuals with different socioeconomic backgrounds.

## Conclusion

The study examined the associations between income and physical activity among Finnish adults. The study extended the previous literature by including self-reported and pedometer-based physical activity in the analysis. Two main findings emerged from the results. First, higher income was associated with higher self-reported leisure-time physical activity for women and men. The results were robust to the inclusion of control variables, as well as the use of a register-based income measure. Second, the pedometer-based results differed by gender: The association was negative or non-existent for men and positive for women. The study suggests that the measurement type of physical activity should be taken into account when possible income effects of physical activity are analyzed and further policy implications proposed.

## Supporting Information

S1 Table Descriptive statistics of the Study Sample and the Full Sample. (DOCX)
