# Supplementary material for: Income and Physical Activity among Adults: Evidence from Self-Reported and Pedometer-Based Physical Activity Measurements
Source: PLoS One. 2015 Aug 28;10(8):e0135651. doi: 10.1371/journal.pone.0135651 (PMC4552741; doi:10.1371/journal.pone.0135651)
Supplement: S1 Table — (DOCX) [file pone.0135651.s001.docx]

# Supporting Information

**S1 Table.** **Descriptive statistics of the Study Sample and the Full Sample.**

| **Variable** | **Study Sample (N=753)** | | **Full Sample** | | | | **P-value^a^** | |
| --- | --- | --- | --- | --- | --- | --- | --- | --- |
|  | **Women** | **Men** | **Women** | | **Men** | | **Women** | **Men** |
|  | **Mean (SD)** | **Mean (SD)** | **Mean (SD)** | **N** | **Mean (SD)** | **N** |  |  |
| **Physical activity** | | | | | | | | |
| Physical Activity Index (PAI)^b^ | 9.36 (1.77) | 8.98 (1.83) | 9.11 (1.87) | 1064 | 8.91 (1.89) | 846 | 0.003 | 0.541 |
| Total Steps / Day | 8865 (2811) | 8101 (2874) | 8318 (3007) | 887 | 7586 (3044) | 633 | <0.001 | 0.003 |
| Aerobic Steps / Day | 2789 (2174) | 2005 (2004) | 2247 (2167) | 887 | 1450 (1904) | 633 | <0.001 | <0.001 |
| **Socioeconomic characteristics** | | | | | | | | |
| Age | 47.75 (4.98) | 41.68 (5.12) | 41.53 (4.99) | 1832 | 41.36 (4.99) | 1764 | 0.318 | 0.302 |
| Income^c^ | 6.72 (2.69) | 8.63 (3.10) | 6.56 (2.80) | 1067 | 8.36 (3.15) | 874 | 0.183 | 0.147 |
| Education (years) | 16.27 (3.31) | 15.5 (3.33) | 15.78 (3.37) | 1226 | 14.84 (3.56) | 998 | 0.001 | 0.001 |
| Work status^d^ | 0.92 (0.28) | 0.98 (0.16) | 0.88 (0.32) | 1226 | 0.96 (0.20) | 1002 | 0.011 | 0.109 |
| Light sedentary work | 0.34 (0.47) | 0.33 (0.47) | 0.30 (0.46) | 1226 | 0.28 (0.45) | 1002 | 0.131 | 0.089 |
| Heavy physical work | 0.01 (0.07) | 0.02 (0.12) | 0.01 (0.07) | 1226 | 0.03 (0.18) | 1002 | 0.808 | 0.018 |
| Number of children | 1.75 (0.44) | 1.69 (0.46) | 1.76 (0.43) | 1228 | 1.68 (0.47) | 987 | 0.397 | 0.708 |
| Married | 0.79 (0.41) | 0.83 (0.38) | 0.77 (0.42) | 1225 | 0.79 (0.41) | 998 | 0.342 | 0.055 |
| Suburb | 0.49 (0.50) | 0.49 (0.50) | 0.48 (0.50) | 1218 | 0.47 (0.50) | 995 | 0.678 | 0.402 |
| **Health status** | | | | | | | | |
| Number of diseases^e^ | 1.13 (1.23) | 0.85 (1.02) | 1.18 (1.31) | 1179 | 0.86 (1.01) | 959 | 0.342 | 0.860 |
| Body Mass Index | 24.76 (4.42) | 26.17 (3.96) | 25.38 (5.06) | 1183 | 26.75 (4.24) | 987 | 0.002 | 0.016 |
| **Family background factors** | | | | | | | | |
| Education (years) Mother | 10.06 (3.05) | 10.27 (3.31) | 9.94 (3.13) | 1774 | 10.04 (3.28) | 1705 | 0.371 | 0.602 |
| Education (years) Father | 9.90 (3.81) | 9.88 (3.79) | 9.71 (3.65) | 1600 | 9.78 (3.66) | 1507 | 0.273 | 0.824 |
| Physical activity Mother^f^ | 1.67 (1.51) | 1.62 (1.48) | 1.64 (1.47) | 1780 | 1.65 (1.49) | 1700 | 0.630 | 0.817 |
| Physical activity Father^f^ | 1.88 (1.70) | 1.81 (1.51) | 1.86 (1.67) | 1601 | 1.81 (1.63) | 1518 | 0.725 | 0.979 |

^a^ P-values for the differences between the study sample and the full sample (T-test). ^b^ Physical Activity Index (PAI), ranging from 5 to 15, is a summary of five variables that illustrates the frequency and the intensity of physical activity, the average duration of physical activity session, the frequency of moderate to vigorous physical activity sessions, and participation in organized sports during leisure time.
^c^ Income categories: 1 = < €5000, 2 = €5000–10000, 3 = €10001–15000, 4 = €15001–20000, 5 = €20001–25000, 6 = €25001–30000, 7 = €30001–35000, 8 = €35001–40000 9 = €40001–45000, 10 = €45001–50000, 11 = €50001–55000, 12 = €55001–60000, 1 3= > €60000. ^d^ Dummy-variable which gets value 1 if working, and value 0 if not working. ^e^ Self-reported number of diseases. ^f^ Self-reported parental physical activity obtained in 1980. The question contained the frequency of physical activity (1= Never, 2 =Once a month, 3 = 2-3 times/month , 4 = Once a week , 5 = 2-6 times/week 6 = Daily).
